# Leprosy

**Published:** 1889-12-21

**Authors:** 


### THE PRACTITIONER'S OUTLOOK AND RETROSPECT.

[*The Editor will be glad to receive offers of co-operation and contributions from members of the profession. There is no doubt that much valuable material full of interest and instruction is at present lost to science. This is largely so in the case of* (1) *the younger and less-known members of the hospital staff,* (2) *those connected with cottage hospitals, and* (3) *the great body of medical practitioners not attached to any institution. The co-operation of all is cordially invited to enable the Editor to make* THE HOSPITAL *of the utmost possible use and value to the profession. Specialists willing to review books on their special subjects are invited to communicate this fact at once. All letters should be addressed to* THE EDITOR, THE LODGE, PORCHESTER SQUARE, LONDON, W.]

### MEDICINE.

### LEPROSY.

Dr. P. S. Abraham publishes a paper entitled "The Etiology of Leprosy—a Criticism on some Current Views" (*Provincial Medical Journal*). The author starts by remarking that, putting aside the bacillus which all pathologists agree to be found in every leprous neuroplasm, the supposed etiological factors of leprosy are (1) heredity, (2) contagion, (3) a diet of fish. As regards heredity, Dr. Abraham quotes Mr. Jonathan Hutchinson, who says, what is perfectly true, that leprosy attacks individuals who have not the slightest hereditary taint. This being admitted, however, we certainly do not perceive how it proves that leprosy is not hereditary. Syphilis may be acquired, but it is also hereditary. Dr. Abraham also quotes Dr. Hansen, who, as the result of a visit to North America to see what had become of Norwegians who had gone there as lepers, came to the conclusion that leprosy is not inherited. Hansen, however, has long held that leprosy is not inherited, and must have made the journey mentioned with preconceived views. Had Dr. Abraham wished, he might have quoted others who hold that leprosy is not inherited. Morehead, in India, did not believe in the hereditaryness of the disease. Lewis and Cuningham, in India, came to the conclusion that heredity could not be a great factor. Carter believes the hereditary taint to be doubtful. But the fact of leper families abounding in India cannot be ignored, notwithstanding that some of the children of lepers escape the disease. As there are various other diseases which are admittedly hereditary, but which do not affect the whole of a family, so we have no right to assume that leprosy is not hereditary because some member of a leper family escape. If leprosy is not hereditary, it is certainly very different from most other constitutional diseases. As regards contagion, Dr. Abraham thinks that although clear and distinct instances of the direct communicability of leprosy from person to person are few and far between, we cannot now doubt its contagiousness. Moreover, Dr. Abraham states that, "with the facts at present at our disposal, it appears to be a pure assumption, unsupported by valid evidence, to say that leprosy can only gain a footing in the human body *per unam viam.*" Acquired syphilis, however, can only be disseminated by direct or indirect contact of syphilitic material, and there are many facts tending to evidence that, for the communication of leprosy, discharge from a leprous sore must come into communication with the abraded skin of a healthy person. With reference to the fish diet theory, Dr. Abraham quotes Mr. Hillis, who wrote, in 1881, that this theory may be now laid aside as obsolete. Still, like many other theories, it reappears again and again. If there is one thing more certain than another, it is that a fish diet is not the cause of leprosy. There is no doubt that the disease prevails, especially in tropical countries, to a very large extent among the fish-eating populations of sea boards. But, on the other hand, it prevails almost to the same extent, in the interior of India, many miles from the coast, where the people never see sea-fish, and very rarely fresh-water fish—probably not eating fish once in a lustrum. Similarly, leprosy has been attributed to want of salt; but it prevails where salt is the produce of the earth, even on the banks of the great Sambhur salt lake, where salt may be had for the asking.

